# Polarization-insensitive GaN metalenses at visible wavelengths

**DOI:** 10.1038/s41598-021-94176-7

**Published:** 2021-07-15

**Authors:** Meng-Hsin Chen, Cheng-Wei Yen, Chia-Chun Guo, Vin-Cent Su, Chieh-Hsiung Kuan, Hoang Yan Lin

**Affiliations:** 1 Department of Electrical Engineering and Graduate Institute of Photonics and Optoelectronics, National Taiwan University, Taipei, 10617 Taiwan; 2 Department of Electrical Engineering and Graduate Institute of Electronics Engineering, National Taiwan University, Taipei, 10617 Taiwan; 3 Department of Electrical Engineering, National United University, Miaoli, 36003 Taiwan

**Keywords:** Nanoscience and technology, Optics and photonics

## Abstract

The growth of wide-bandgap materials on patterned substrates has revolutionized the means with which we can improve the light output power of gallium nitride (GaN) light-emitting diodes (LEDs). Conventional patterned structure inspection usually relies on an expensive vacuum-system-required scanning electron microscope (SEM) or optical microscope (OM) with bulky objectives. On the other hand, ultra-thin metasurfaces have been widely used in widespread applications, especially for converging lenses. In this study, we propose newly developed, highly efficient hexagon-resonated elements (HREs) combined with gingerly selected subwavelength periods of the elements for the construction of polarization-insensitive metalenses of high performance. Also, the well-developed fabrication techniques have been employed to realize the high-aspect-ratio metalenses working at three distinct wavelengths of 405, 532, and 633 nm with respective diffraction-limited focusing efficiencies of 93%, 86%, and 92%. The 1951 United States Air Force (USAF) test chart has been chosen to characterize the imaging capability. All of the images formed by the 405-nm-designed metalens show exceptional clear line features, and the smallest resolvable features are lines with widths of 870 nm. To perform the inspection capacity for patterned substrates, for the proof of concept, a commercially available patterned sapphire substrate (PSS) for the growth of the GaN LEDs has been opted and carefully examined by the high-resolution SEM system. With the appropriately chosen metalenses at the desired wavelength, the summits of structures in the PSS can be clearly observed in the images. The PSS imaging qualities taken by the ultra-thin and light-weight metalenses with a numerical aperture (NA) of 0.3 are comparable to those seen by an objective with the NA of 0.4. This work can pioneer semiconductor manufacturing to choose the polarization-insensitive GaN metalenses to inspect the patterned structures instead of using the SEM or the bulky and heavy conventional objectives.

## Introduction

As one of the most promising next-generation semiconductors, wide-bandgap gallium nitride (GaN) has recently drawn much attention due to its widespread applications in high-power electronics^1,2^, high-frequency devices^3,4^, blue light-emitting diodes (LEDs)^5,6^, laser diodes^7–9^, and so on. Among these applications, the blue LEDs as excitation sources of white LEDs have revolutionized lighting technology in the visible. These white LEDs have been served as state-of-the-art solid-state lighting^10–12^. The blue LEDs are typically grown on micro/nano-sized patterned-sapphire substrates (PSSs)^13–17^ owing to challenges in narrow photon escape cone leading to low light-extraction efficiency (LEE)^18^ of the LEDs as well as high threading dislocations caused by the hetero-epitaxial growth. Such PSSs are usually examined by a scanning electron microscope (SEM) system of high cost and low speed or an optical microscope (OM) that requires bulky and heavy objectives. There is a need to develop ultra-light and thin optics to replace current conventional objectives.

Metasurfaces^19–22^ are typically composed of near-wavelength artificial patterns separated by subwavelength distances, making it possible to achieve high-performance ultrathin optics. Early demonstrations of practical applications have been realized by using reflective metasurfaces^23–25^, which are made of noble metals resulting in low transmission efficiency in the visible regime. On the other hand, the rapid development of all-dielectric metasurfaces^26–31^ operating in transmissive mode can be ascribed to the high intrinsic losses of plasmonic metals in the visible spectrum. Besides, one of the significant applications for the development of dielectric metasurfaces is well-known as metalenses.

Recently, highly efficient metalenses constructed with a Pancharatnam-Berry (PB) phase rotational morphology have been successfully manufactured using either top-down or bottom-up approaches depending on the fabrication processes with which the artificial patterns are made^32–37^. However, the metalenses based on the PB-phase method require circularly polarized light as an excitation incidence to reach their full-phase control. Although this obstacle can be circumvented by using nanostructures with symmetric cross-sections, polarization-insensitive metalenses^38–41^ of high performance for the desired wavelength require the development of highly efficient building blocks along with carefully selected subwavelength periods and well-established fabrication processes.

This work demonstrates three distinct polarization-insensitive metalenses of high performance at wavelengths of 405, 532, and 633 nm with experimentally diffraction-limited focusing efficiencies of 93%, 86%, and 92%, respectively. The building blocks of the metalenses are the so-called high-aspect-ratio GaN hexagon-resonated elements (HREs) with an axis of six-fold rotational symmetry. The carefully opted subwavelength periods of the elements combined with the matured top-down manufacturing enable fabrication of these high-aspect-ratio GaN HREs with nearly vertical sidewalls. The 1951 United States Air Force (USAF) test chart has been served as our imaging target to characterize imaging capability for the fabricated metalenses. The results show that the metalenses with a numerical aperture (NA) of 0.3 can resolve nano-sized features as small as 870 nm in the 1951 USAF sample.

Moreover, few studies have focused on metalenses applied to the examination of patterned substrates used for the growth of the GaN-based LEDs^42^. We have prepared a commercially available PSS being cautiously inspected by a high-resolution SEM system for the proof of concept. A mass-produced objective (20 × Olympus; NA = 0.4) has also been chosen to compare the native PSS imaging performance of our metalenses with the objective. Although our metalenses are designed at the NA of 0.3, the PSS imaging qualities are comparable or even superior to those formed by the objective. For the practical application, the GaN LED epitaxial layers with multiple quantum wells (MQWs) emitting at a wavelength of ~ 440 nm have been grown on the PSS to be further inspected with the metalens designed at a wavelength of 450 nm. The results show that patterned structures in the epi-wafer can be clearly identified using the 450-nm-designed metalens and the commercialized objective.

### Metalenses design and fabrication

**Figure 1:**
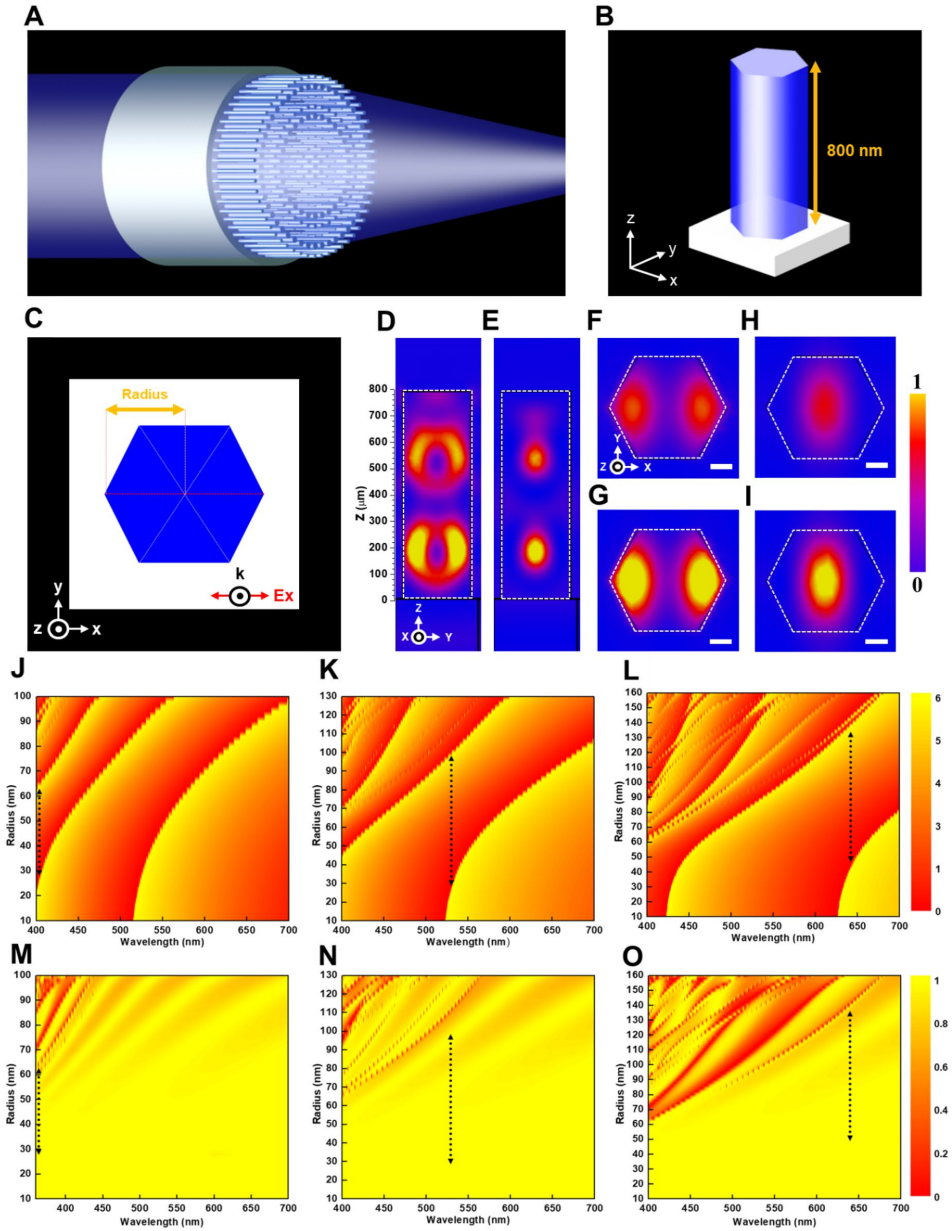
Design and simulation of the building block for metalenses. (**A**) Schematic of a dielectric metalens operating in a transmissive mode. (**B**, **C**) Schematics of the tilt-view and top-view of the building block for the metalens composed of the HREs. (**D**, **E**) Simulated electric and magnetic field intensity distribution. (**F**, **G**) The x–y cross-sections of simulated electric field intensity distribution at z = 500 and 200 µm, individually. (**H**, **I**) The x–y cross-sections of simulated magnetic field intensity distribution corresponding to (**F**, **G**). Scale bar, 50 nm in (**F**–**I**). (**J**–**L**) Diagrams showing the range of phase modulation for the building block periods of 200, 260, and 320 nm on the combinations of wavelength and radius profiles for the metalenses designed at wavelengths of 405, 532, and 633 nm, respectively. (**M**–**O**) Simulated efficiencies of the metalenses designed at the wavelengths of 405, 532, and 633 nm, respectively. The black dashed lines represent the implemented radius range for the metalens.

Novel optical metalenses are known to promise light-weight and ultra-thin benefits as compared to bulky conventional converging lenses that impede their practical applications. Figure 1A shows a schematic of a dielectric metalens operating in a transmissive mode, and Fig. 1B, C, are respectively the schematics for illustrating the tilt-view and top-view of building blocks of the metalens consisting of the HREs, with linearly x-polarized incident light. The associated phase profile of the metalens^32,33^ should follow as

1 $$\varphi (x,y) = \, - \left( { \, \sqrt {f^{2} + x^{2} + y^{2} } - \, f} \right)\frac{2\pi }{\lambda }$$

where $f$ is the focal length, $x$ and $y$ are coordinates for each HRE, and $\lambda$ is the design wavelength in free space. To maximize transmission efficiency that meets the phase retardation distributions requirements for the metalenses, we choose the HREs made of wide bandgap GaN as being truncated waveguides that eliminate coupling between neighbors by highly concentrating electric and magnetic energy inside the nanostructures as near-field distributions shown in Fig. 1D, E, respectively, with a HRE radius of 140 nm and the 320-nm-period unit cell. The corresponding horizontal cut of the electric energy intensity at z = 500 and 200 µm are respectively revealed in Fig. 1F, G. The associated cross-sectional magnetic energy distributions are also shown in Fig. 1H (z = 500 µm) and I (z = 200 µm). As shown in the figures, such structures can also generate strong electric energy around corners of each HRE (Fig. 1F, G) and converge magnetic energy inside the HRE (Fig. 1H, I)^30,31^. Thus, a large phase compensation of high efficiency can be achieved by using the high-aspect-ratio GaN HREs that allow for strong symmetrical near-field coupling among the corners of the hexagon-shaped nanostructures in addition to excitation of high-order resonances of a waveguide-like cavity inside the nanostructures.

Three periods for the building blocks of the metalenses have been gingerly selected as 200, 260, and 320 nm at the respective design wavelengths of 405, 532, and 633 nm. Simulations using the CST Microwave Studio based on a finite integration time (FIT) method have been employed to calculate phase responses for GaN HREs with various radii and incident wavelengths as individually revealed in Fig. 1J–L, with the corresponding efficiency diagrams of GaN HREs found in Fig. 1M–O. The black dashed lines in Fig. 1J–O, indicate the radii used for the construction of the metalenses in this work. The structural parameters of each building block have been optimized to achieve the highest operating efficiency. It is well noted that those metalenses constructed of the high-aspect-ratio HREs with nearly vertical sidewall require the aforementioned building block periods (p = 200, 260, and 320 nm) carefully chosen. This is because the longer length of the p is, the broader range of radius variation the HRE is needed to achieve 2π phase modulation. Consequently, achieving these vertical-sidewall HREs with smaller or larger radii becomes more challenging because of inevitable lateral etching and smaller spaces between nanostructures. From another point of view, reducing the period of the building blocks results in a less obvious difference between the HRE radii selected for metalens construction. Such smaller periods lead to pronounced fabrication errors while making metalenses due to the stringent fabrication tolerance.

**Figure 2:**
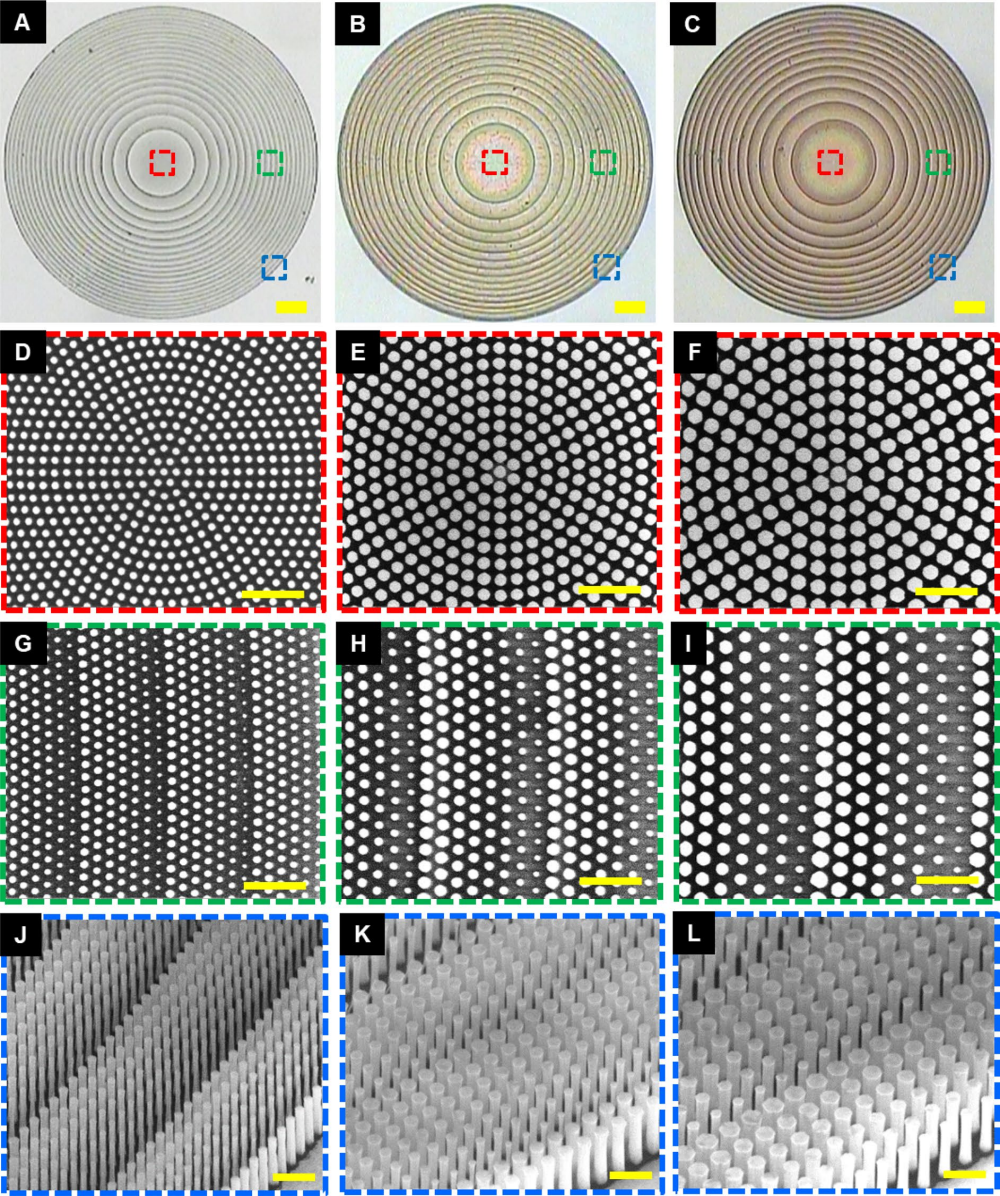
Micrographs of the metalenses. (**A**–**C**) Optical images of the fabricated metalenses designed at wavelengths of (**A**) 405, (**B**) 532, and (**C**) 633 nm. Scale bar: 10 μm. (**D**–**I**) The top-view SEM images shown in (**D**, **G**), (**E**, **H**), and (**F**, **I**) corresponding to the highlighted regions in (**A**–**C**). Scale bar, 2 μm in (**D**–**I**). (**J**–**L**) The tilt-view SEM images shown in (**J**–**L**) corresponding to the highlighted regions in (**A**–**C**). Scale bar, 1 μm in (**J**–**L**).

Figure 2A–C, represent the OM images for three distinct metalenses fabricated at the respective design wavelengths of 405, 532, and 633 nm. The detailed fabrication processes can be found in the Methods section and schematically illustrated in Supplementary Fig. S1. All of the metalenses possess the same NA of 0.3 resulting from the use of a diameter of 100 µm and a focal length of 150 µm. The top-view of SEM micrographs of the metalenses are demonstrated in Fig. 2D–I, and the associated tilt-view SEM images are presented in Fig. 2J–L. With the carefully selected periods together with the well-developed semiconductor manufacturing, the GaN HREs with nearly vertical sidewalls can be successfully fabricated to accomplish the high-aspect-ratio metalenses by using the top-down fabrication approach. We highly note that it is very challenging without gingerly choosing a designed period for the building block of a metalens at visible wavelengths.

### Experimental measurements

**Figure 3:**
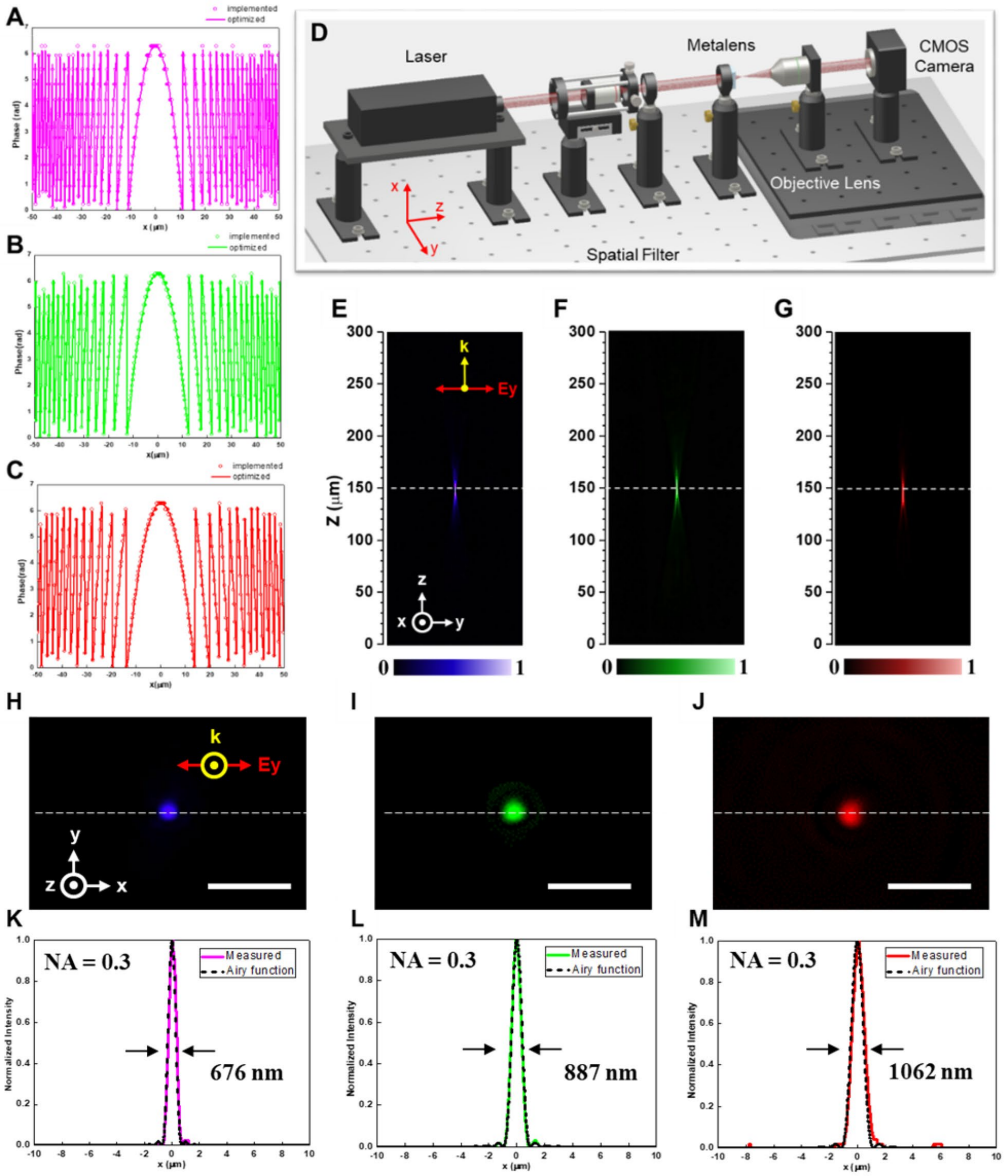
Characterization of the metalenses. (**A**–**C**) Phase profiles for the metalenses designed at wavelengths of (**A**) 405, (**B**) 532, and (**C**) 633 nm. Solid lines depict optimized phase profiles, and hollow circles indicate implemented phase profiles. (**D**) Measurement setup for metalenses characterization. (**E**–**G**) The intensity profiles along the axial planes for the metalenses designed at wavelengths of (**E**) 405, (**F**) 532, and (**G**) 633 nm. (**H**–**J**) Measured focal spots of the metalenses designed at wavelengths of (**H**) 405, (**I**) 532, and (**J**) 633 nm. Scale bar: 6 μm. (**K**–**M**) Corresponding horizontal cuts of focal spots with the dashed lines referring to normalized ideal Airy function.

The required phase profiles of the three distinct metalenses are shown as the solid lines in Fig. 3A–C, in which hollow circles indicate the simulated phases associated with the HRE radii used for the realization of the fabricated metalenses. As shown in the figures, the solid lines can be well implemented with the circles for all designs. The experimental setup for measuring intensity profiles along axial planes, focal spots, and efficiencies is schematically displayed in Fig. 3D. These measurements use a laser beam to go through a spatial filter to produce a clean Gaussian beam, which is then focused by the metalenses to generate a highly concentrated spot. Subsequently, the focal spot is collected by an objective lens, followed by capturing an image on a CMOS camera.

Figure 3E–G, perform the intensity profiles along the axial planes for the three distinct metalenses using diode lasers as incident light sources with linear polarization in the y-direction. Here, the objective lens and the CMOS camera are successively moved together along laser light propagation direction to capture images at different x–y planes, which are stitched together to acquire the light intensity distribution for the y–z plane. As revealed in the figures, the brightest focal spots for the three distinct metalenses remain at the designed focal length of 150 μm. The correlated focal spots for the metalenses are demonstrated in Fig. 3H–J, showing highly symmetric profiles and the excellent ability for light convergence. Figure 3K–M, further provide the cross-sectional distributions (colorful solid lines) for the measured focal spots, which are extremely close to respective normalized Airy functions as black-dashed lines. The measured full-widths at half-maximum (FWHMs) of the metalenses at the wavelengths of 405, 532, and 633 nm correspond to 676, 887, 1062 nm. All of the measured FWHMs are very close to theoretically diffraction-limited values calculated by $\frac{\lambda }{2NA}$, demonstrating superior diffraction-limited focusing capability. Furthermore, focusing efficiencies for the metalenses are experimentally measured as high as 93% (405 nm), 86% (532 nm), and 92% (633 nm). The efficiency is defined as the ratio of transmitted optical power at diffraction-limited focusing to the optical power of the incident beam passing through a pinhole with the same diameter of the fabricated metalenses.

As demonstrated in Supplementary Fig. S2, we also simulated cross-sectional intensity profiles for the metalenses with the same NA of 0.3 but with a smaller focal length of 12 μm due to our computational limitation. The simulated efficiencies for the metalenses at wavelengths of 405, 532, and 633 nm are respectively 95%, 90%, and 93%. It is also noted that the fabrication imperfection in the hexagon-shaped GaN nanostructures with smaller radii lead to the optical performance to deviate from the simulation results. Moreover, the polarization insensitivity for all of the fabricated metalenses still needs to be carefully examined. As a consequence, both left-handed circularly polarized (LCP) and right-handed circularly polarized (RCP) laser beams^43–45^ have been utilized as incident sources for the metalenses to measure the corresponding intensity profiles and diffraction-limited focal spots as demonstrated in Supplementary Fig. S3. Light intensity distribution for all of the metalenses with LCP and RCP incident laser light remains the same, and negligible change in the measured FWHMs can be observed in the figures. The experimental results show that these metalenses are polarization-insensitive and are capable of focusing light into diffraction-limited spots of high efficiency. We attribute extraordinary optical properties of the metalenses to the matured fabrication techniques combined with the gingerly chosen period of the building blocks composed of highly efficient HREs.

**Figure 4:**
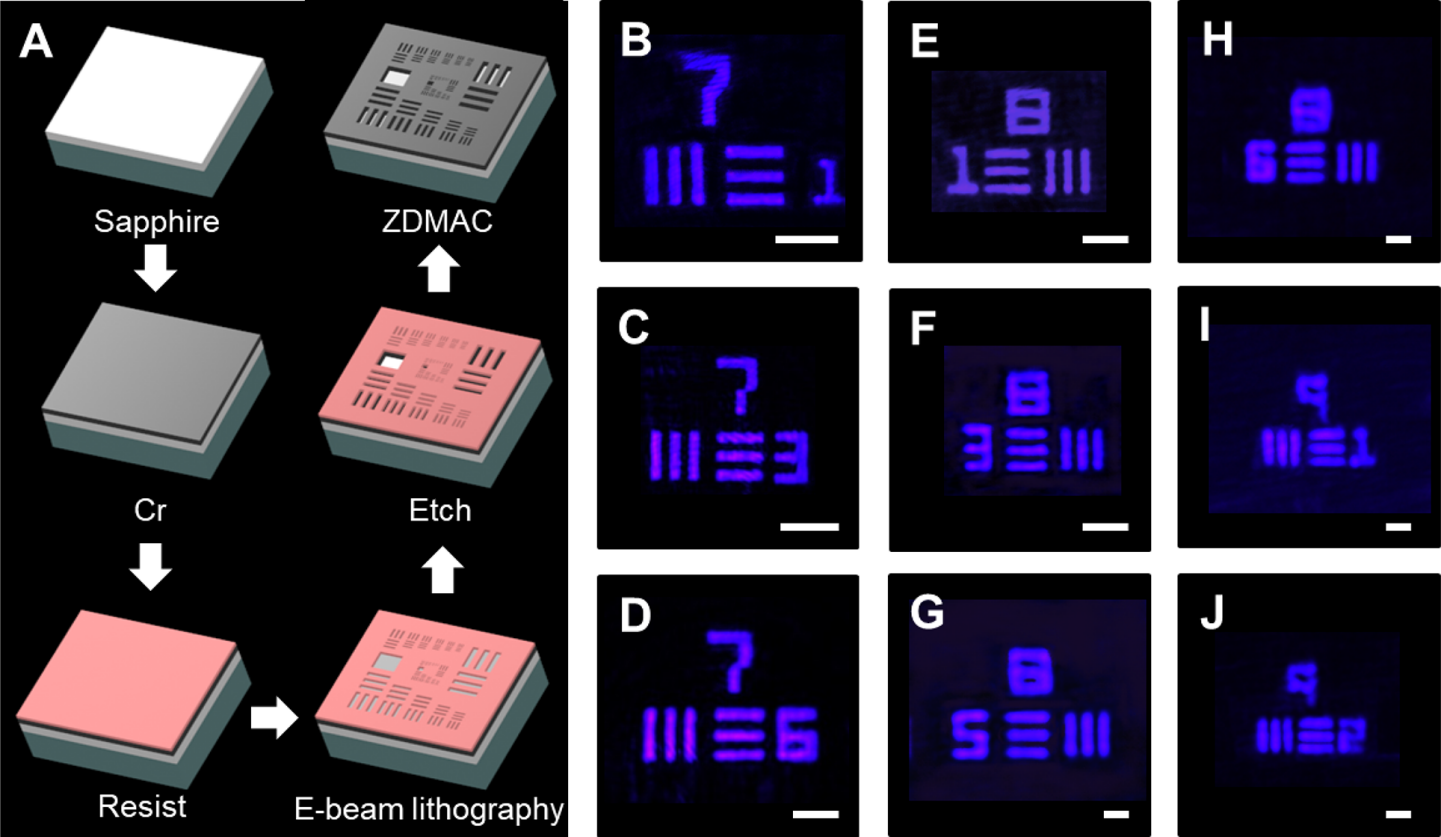
Imaging with the metalenses. (**A**) Fabrication of the 1951 USAF resolution test chart. (**B**–**J**) Images of the 1951 USAF resolution test chart formed by the metalenses at the laser wavelength of 405 nm. The smallest features are (**B**) line widths of 3.91 µm and center-to-center distances of 7.82 µm, (**C**) line widths of 3.1 µm and center-to-center distances of 6.2 µm, (**D**) line widths of 2.19 µm and center-to-center distances of 4.38 µm, (**E**) line widths of 1.95 µm and center-to-center distances of 3.9 µm, (**F**) line widths of 1.55 µm and center-to-center distances of 3.1 µm, (**G**) line widths of 1.23 µm and center-to-center distances of 2.46 µm, (**H**) line widths of 1.1 µm and center-to-center distances of 2.2 µm, (**I**) line widths of 0.98 µm and center-to-center distances of 1.96 µm, and (**J**) line widths of 0.87 µm and center-to-center distances of 1.74 µm. Scale bar, 20 μm in (**B**, **C**). Scale bar, 10 μm in (**D**–**F**). Scale bar, 3 μm in (**G**–**J**).

One practical application for metalenses is imaging. Here we have selected the 1951 USAF as our imaging target illuminated by laser light beams at three distinct wavelengths. Nevertheless, a commercially available USAF sample only provides the smallest width of lines that are resolvable for 2.19 μm. Therefore, a USAF sample with smaller features is fabricated and carefully examined by the high-resolution SEM system. A schematic for the fabrication processes of the imaging target is illustrated in Fig. 4A. The fabrication starts with evaporating a chromium (Cr) layer on a sapphire substrate deposited with a thin silicon dioxide (SiO_2_) layer, which is subsequently coated by a resist layer followed by exposing an electron beam directly onto the resist-coated substrate. The subsequent process builds up 1951 USAF patterns by etching the Cr layer after developing the exposed resist-coated substrate. Details of the measurement configuration are shown in Supplementary Fig. S4.

Figure 4B–J, show the images taken by the 405-nm-designed metalens with a NA of 0.3. As exhibited in the figures, all of which exhibit extreme clear line features, and the smallest features we can observe are line with widths of 870 nm, where the features are separated by a center-to-center distance of 1.74 μm. On the contrary, in Supplementary Fig. S5, the line features in images formed by the metalens designed at the wavelength of 532 nm are clearly resolvable until approaching the line widths of 0.98 μm (Fig. S5H). Nevertheless, it is still predictable that the same line features captured by the metalens designed at the wavelength of 633 nm turn into the blurred one and cannot be distinguished as revealed in Fig. S5Q due to the larger diffraction-limited value of the metalens. As a result, the demonstration in clearly resolving line features corresponding to the distinct diffraction-limited metalenses can be ascribed to the successful development of the highly-efficient HREs, the gingerly chosen subwavelength periods together with the triumphant fabrication techniques.

**Figure 5:**
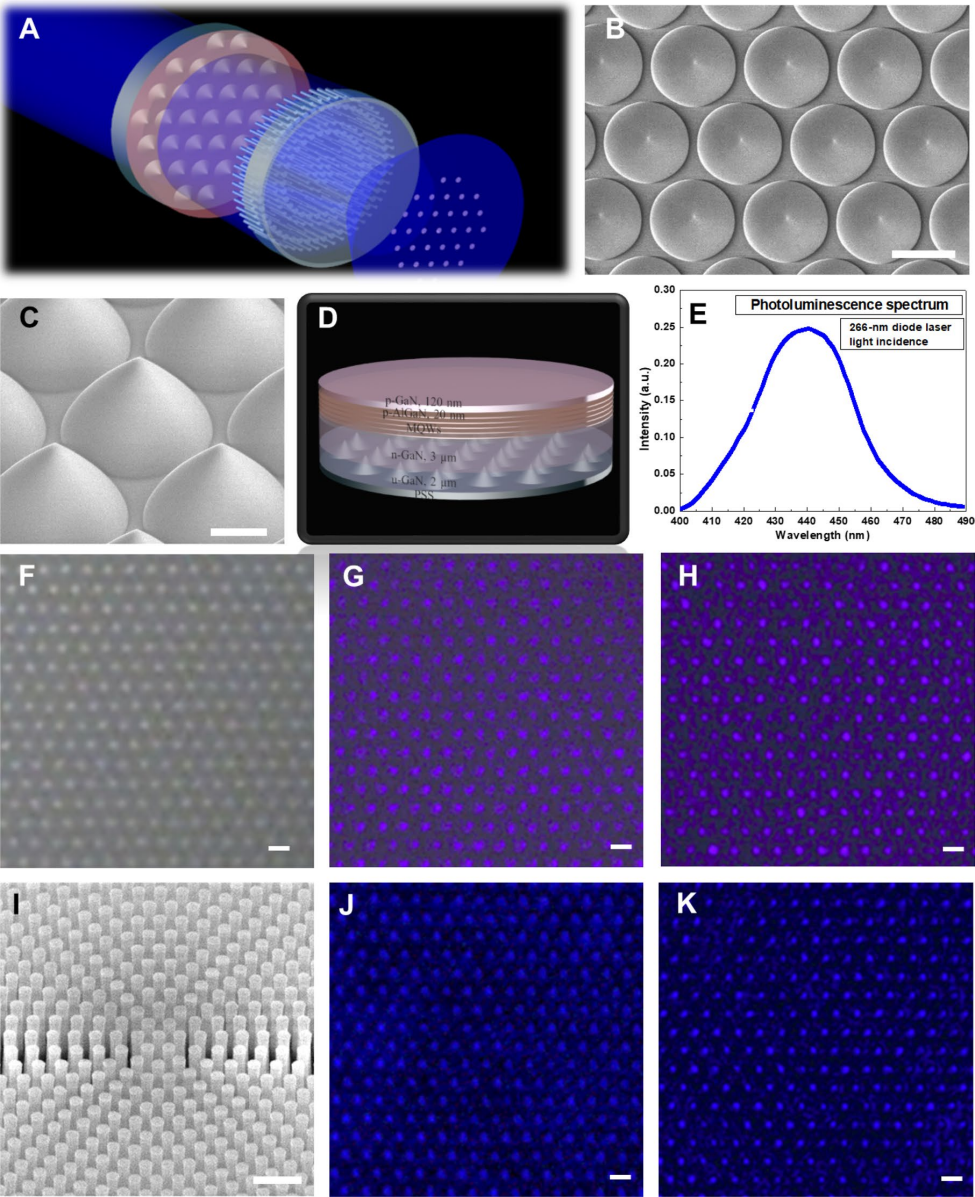
Imaging with the native PSS and the blue LED on the PSS. (**A**) Schematic of patterned structures inspected by the metalens. (**B**) The top-view SEM micrograph of the native PSS. Scale bar: 2 μm. (**C**) The tilt-view SEM micrograph of the PSS. Scale bar: 1 μm. (**D**)The epitaxial structures of the blue LED. (**E**) Photoluminescence spectrum. Images of the PSS formed by the objective lens (NA = 0.4) with the incidences of (**F**) the halogen lamp and (**G**) the 405-nm laser light. (**H**) PSS Image formed by the 405-nm-designed metalens (NA = 0.3) at the laser wavelength of 405 nm. Scale bar, 3 μm in (**F**–**H**). (**I**) The tilt-view SEM micrograph of the fabricated metalens designed at the wavelength of 450 nm. Scale bar: 500 nm. Images of the PSS on which the blue LED taken by (**J**) the objective lens (NA = 0.4) at the laser wavelength of 450 nm, and (**K**) the 450-nm-designed metalens (NA = 0.3) at the laser wavelength of 450 nm. Scale bar, 3 μm in (**J**, **K**).

The differences in refractive indexes and lattice constants between sapphire and GaN severely limit the performance of GaN-based LEDs. The use of PSSs has been demonstrated so far to show benefits in increasing LEE and reducing the threading dislocations for the LEDs. In order to avoid the expensive vacuum system required for SEM and bulky conventional objectives, the metalens at the design wavelength of 405 nm has been utilized to inspect feature sizes and morphology of the fabricated PSSs as shown schematically in Fig. 5A. The commercially available PSS constructed of dome-shaped posts has been prepared to serve as the inspection target. The center-to-center distance of the posts in the PSS is 3 μm. Each dome-shaped post has a height of 1.65 μm and a bottom width of 2.8 μm as top-view and tilt-view SEM images demonstrated in Fig. 5B, C, respectively. In addition to the dome-shaped PSS, the growth of GaN-based blue LED epitaxial layers emitting blue light at a peak wavelength of ~ 440 nm on the PSS has been prepared by using the metal–organic chemical vapor deposition (MOCVD) system as the schematic depicted in Fig. 5D, with the detailed epitaxial conditions found in the Methods section. To verify the peak emission wavelength for the blue LED on the PSS, the photoluminescence spectrum was carried out by a diode laser at a wavelength of 266 nm as an excitation light source, as shown in Fig. 5E. The blue LED grown on the PSS emits light at the peak wavelength of ~ 440 nm. The measured result for the peak emission wavelength of the blue LED on the PSS is in accordance with our design.

The setup for the experimental measurement is the same as one shown in Supplementary Fig. S4. except that the 1951 USAF has been replaced by the commercialized PSS. Figure 5F, G, demonstrate PSS images formed by the mass-produced objective (20 × Olympus; NA = 0.4) with light incidences of a halogen lamp and 405-nm diode laser, respectively. The image captured by the 405-nm-designed metalens is shown in Fig. 5H. As compared in figures, the metalens with the smaller NA of 0.3 can also provide a clear and high contrast image for the summits of the dome-shaped posts in the PSS. This is understood because the commercialized objective is designed for the broadband operation, resulting in undesirable wavefront aberration for a light incidence at a specific wavelength. However, it is worth noting that a fair inspection index can be developed in the future to compare the imaging performance of the metalenses and the conventional objective.

Another benefit of using the metalens is the capability to examine the PSSs even with the GaN-based blue LED epitaxial layers on them, which is unattainable for the vacuum-required SEM system. As shown in Fig. 5E, the MQWs of the blue LED is verified to emit blue light at a wavelength of ~ 440 nm. Therefore, we fabricated another metalens working at the wavelength of 450 nm with the same focal length of 150 μm and NA = 0.3. The top-view SEM micrographs of the metalens are presented in Fig. 5I. Unsurprisingly, the one taken by the objective shown in Fig. 5J, is vaguer than the image pictured by the 450-nm-designed metalens, as verified in Fig. 5K, which can clearly and sharply visualize the post apexes in the PSS. The results show that the PSS for the growth of the GaN-based LED can be inspected by the metalens with an appropriately designed wavelength. In addition, this work provides opportunities for the PSS manufacturing companies to start to use metalens to examine fabricated structures instead of using the bulky conventional objectives.

## Conclusion

In this work, we have successfully developed the high-aspect-ratio HREs of high efficiency as the building blocks for the metalens construction. The polarization-insensitive metalenses of high performance can be realized with gingerly chosen subwavelength periods and well-developed fabrication. The metalenses are capable of focusing light into the diffraction-limited focal spots and provide extremely clear imaging for the line widths as small as 870 nm. For the proof of concept, the imaging capability for capturing the commercial PSS is also demonstrated. The metalenses with the smaller NA of 0.3 can even perform superior contrast and clearer images compared to that taken by the mass-produced objective with the NA of 0.4. This work demonstrates the tremendous capacity for the ultra-thin and light-weight metalenses being used for fabricated structure inspection of the patterned substrates and offers another novel and advanced optics based on semiconductor manufacturing.

## Methods

### Details of fabrication parameters and processes

A metal–organic chemical vapor deposition (MOCVD) system has been utilized for the growth of an 800-nm-thick gallium nitride (GaN) layer on a sapphire substrate, followed by depositing a 400-nm-thick silicon dioxide (SiO_2_) layer using plasma-enhanced chemical vapor deposition (PECVD). The distinct metalens patterns in the resist layer have been created by using the electron-beam lithography after spin-coating a 100-nm-thick resist (ZEP520A) layer on the SiO_2_-deposited substrate. Afterward, a 40-nm-thick chromium (Cr) layer is evaporated on the developed substrate, and a lift-off process is implemented. The subsequent process is to transfer the patterns from the patterned Cr layer as a hard mask to the SiO_2_ layer by reactive-ion etching (RIE). The remaining Cr layer is removed with the CR-7 etchant, and then the GaN layer is dry-etched by inductively coupled plasma reactive-ion etching (ICP-RIE). Finally, the sample is accomplished after dipping the fabricated substrate in the buffered oxide etch (BOE) solution.

### The epitaxial structures of the blue LED

The MOCVD system was also employed for the growth of GaN-based blue LED epitaxial layers on a commercialized patterned sapphire substrate (PSS). Prior to the growth, the PSS was thermally baked at 1130 °C in hydrogen gas to remove surface contamination. Then, a low-temperature GaN nucleation layer was grown at 550 °C. Subsequently, the reactor temperature was raised to 1130 °C for the growth of a 2-µm-thick undoped GaN layer and a 3-µm-thick Si-doped n-type GaN layer. Then, five pairs of InGaN/GaN MQWs with a 2.8-nm-thick InGaN well and a 10-nm-thick GaN barrier were deposited, followed by a 20-nm-thick p-AlGaN electron blocking layer and a 120-nm-thick Mg-doped p-type GaN layer. The InGaN/GaN MQWs were designed to emit blue light at a peak wavelength of ~ 440 nm.

## Supplementary Information

Supplementary Information.

## Data Availability

The data regarding the findings of this study are available from the corresponding authors.
