# Supplementary material for: Polarization-insensitive GaN metalenses at visible wavelengths
Source: Sci Rep. 2021 Jul 15;11:14541. doi: 10.1038/s41598-021-94176-7 (PMC8282812; doi:10.1038/s41598-021-94176-7)
Supplement: Supplementary file 1 — Supplementary Information. [file 41598_2021_94176_MOESM1_ESM.docx]

**Supplementary Materials for**

**Polarization-insensitive GaN metalenses at visible wavelengths**

Meng-Hsin Chen^1^, Cheng-Wei Yen^2^, Chia-Chun Guo^3^, Vin-Cent Su^3†^, Chieh-Hsiung Kuan^1,2†^, and Hoang Yan Lin^1†^

*^1^Department of Electrical Engineering and Graduate Institute of Photonics and Optoelectronics, National Taiwan University, Taipei 10617, Taiwan.*

^2^*Department of Electrical Engineering and Graduate Institute of Electronics Engineering, National Taiwan University, Taipei 10617, Taiwan*

^3^*Department of Electrical Engineering, National United University, Miaoli 36003, Taiwan*

†Corresponding authors: [hoangyanlin@ntu.edu.tw](mailto:hoangyanlin@ntu.edu.tw) ; [chkuan@ntu.edu.tw](mailto:chkuan@ntu.edu.tw) ; [vcsu@nuu.edu.tw](mailto:vcsu@nuu.edu.tw)

**Sample fabrication**

**A schematic for the processes of the metalenses**

The schematic figure of the fabrication processes is shown in Fig. S1.


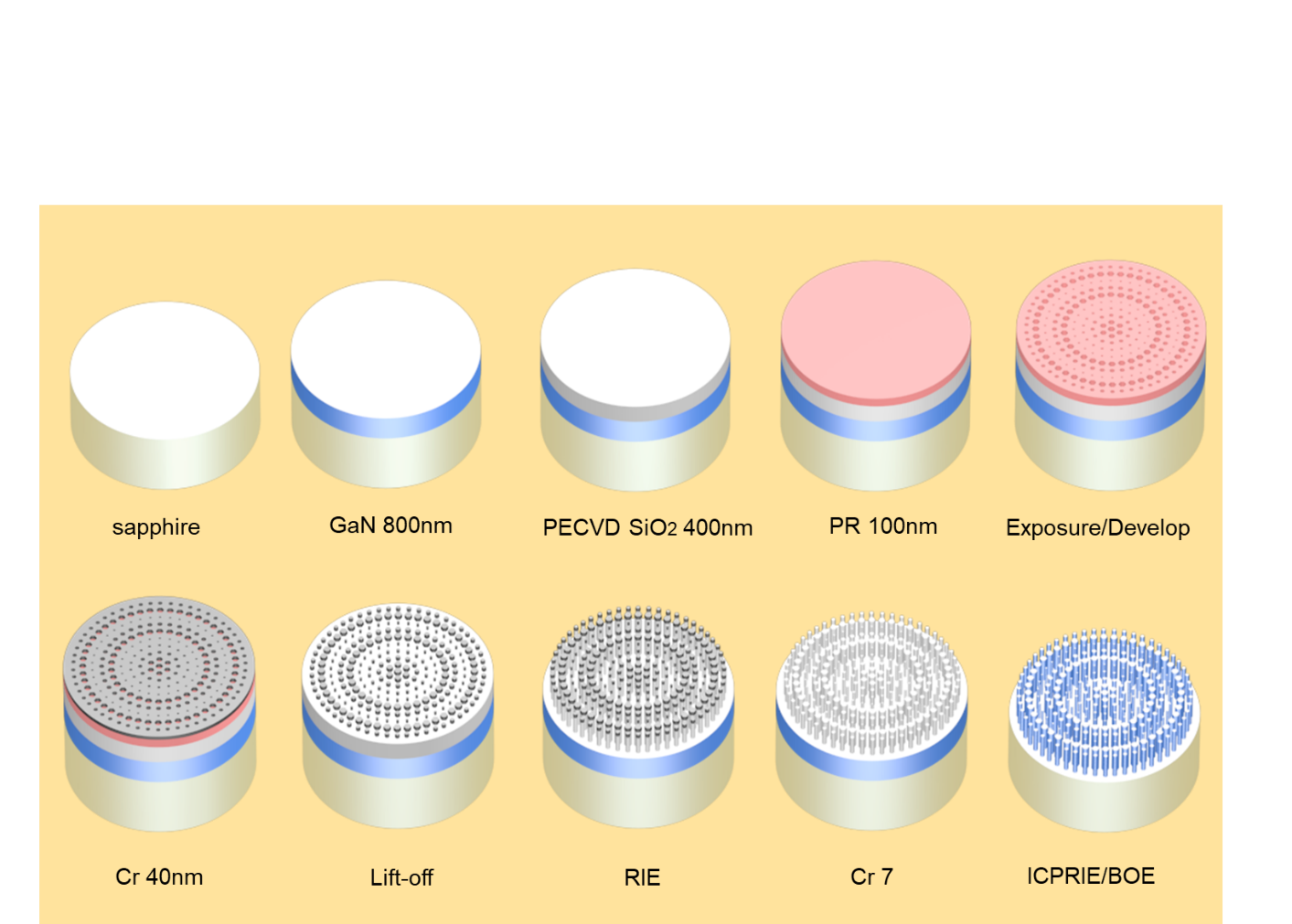


**Fig. S1: The process flow for fabricating metalenses.**

**Experiments**

**Small-diameter metalenses simulation**

The simulation results for metalenses with a smaller diameter of 8 μm and a focal length of 12 μm, yielding the same NA of 0.3, are demonstrated here. The cross-sectional profiles for the metalenses at the wavelengths of 405, 532, 633 nm are shown in Fig. S2, A to C, respectively.


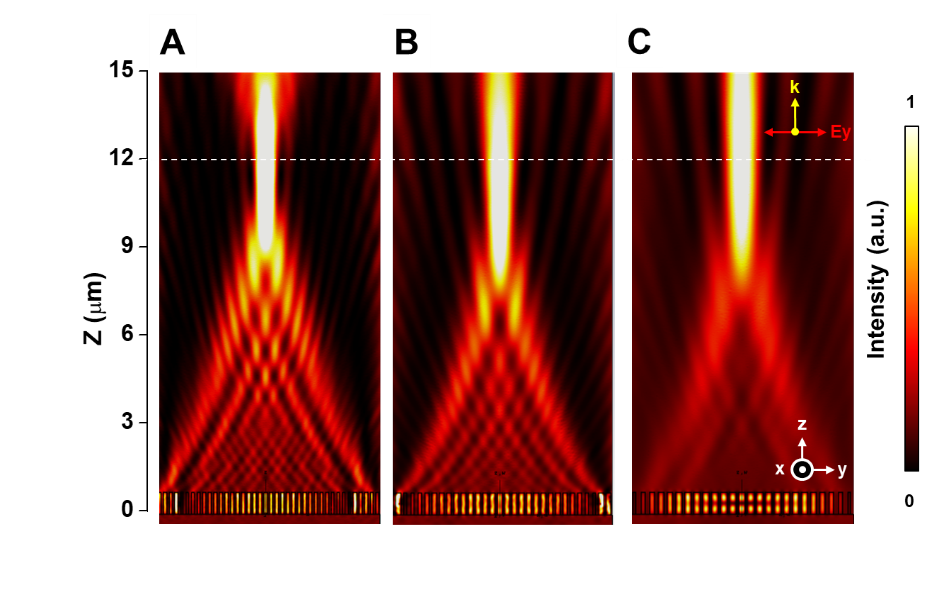


**Fig. S2: Simulated Intensity profiles of the metalenses.**

**Polarization insensitivity measurements**

Both left-handed circularly polarized (LCP) and right-handed circularly polarized (RCP) laser beams have been utilized as incident sources for the metalenses to measure the corresponding intensity profiles (Fig. S3, A to F) and the horizontal cut of the diffraction-limited focal spots (Fig. S3, G to L). Light intensity distribution for all of the metalenses with LCP and RCP laser incident light remains the same, and minor changes in the measured FWHMs can be observed in the figures.


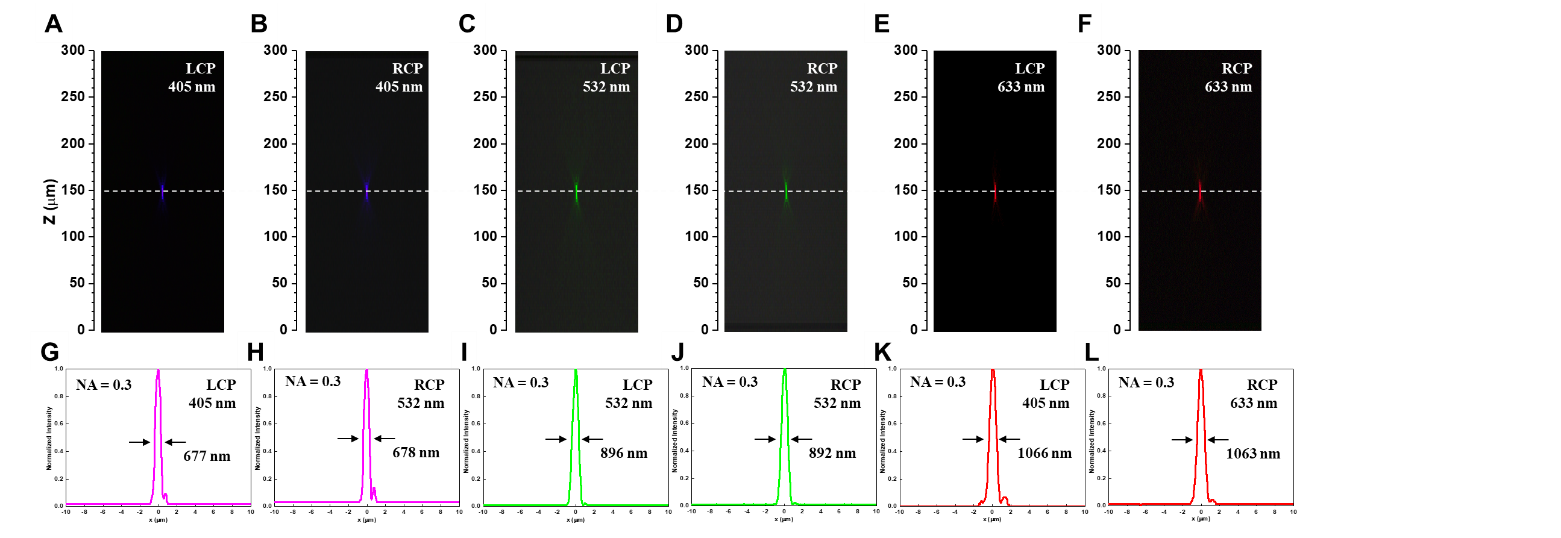


**Fig. S3: Intensity profiles measured along axial planes and corresponding horizontal cuts of focal spots for the RCP and LCP incident laser beams of the metalens designed at the wavelengths of 405, 532, and 633 nm.**

**The 1951 United States Air Force (USAF) resolution test chart measurements**

The laser beam first passes through a spatial filter and a plano-convex lens to form higher-quality laser light. A 10x objective lens is successively used to focus the light onto the USAF 1951 target. A 20x objective lens is used to image the light focused by a metalens. The distance between the 20x objective lens and the metalens can be adjusted by using an electric stage. Finally, a CMOS camera is used to record an image of the 1951 USAF resolution test chart.


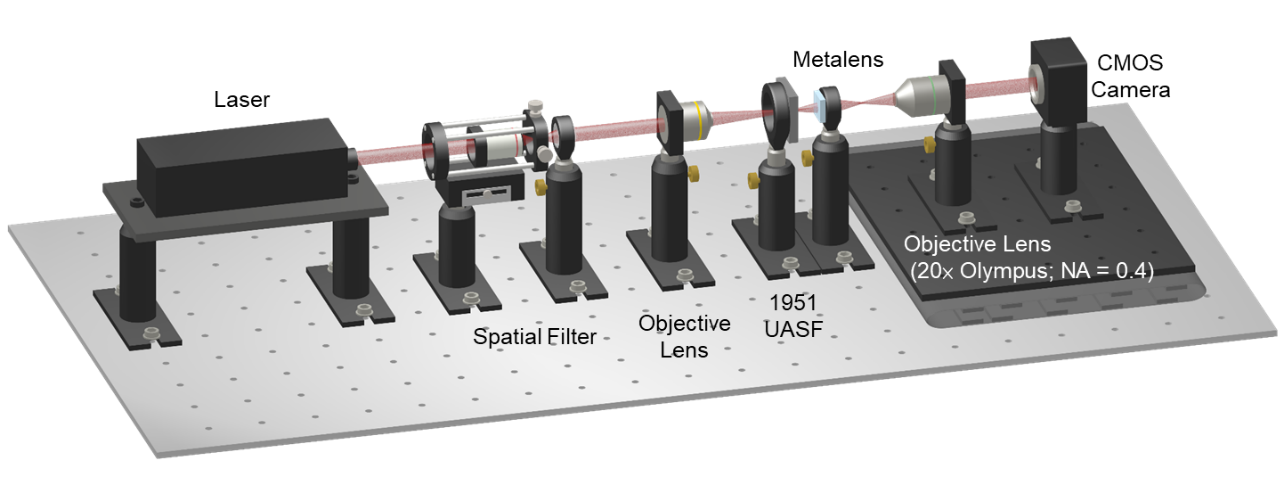


**Fig. S4: The experimental setup for imaging.**

**Imaging with the metalenses at the laser wavelengths of 532 nm and 633nm**

Figure S5 shows the images of the 1951 USAF resolution test chart formed by the metalenses at the laser wavelengths of (A to I) 532 nm and (J to R) 633 nm. The smallest features are (A, J) line widths of 3.91 µm and center-to-center distances of 7.82 µm, (B, K) line widths of 3.1 µm and center-to-center distances of 6.2 µm, (C, L) line widths of 2.19 µm and center-to-center distances of 4.38 µm, (D, M) line widths of 1.95 µm and center-to-center distances of 3.9 µm, (E, N) line widths of 1.55 µm and center-to-center distances of 3.1 µm, (F, O) line widths of 1.23 µm and center-to-center distances of 2.46 µm, (G, P) line widths of 1.1 µm and center-to-center distances of 2.2 µm, (H, Q) line widths of 0.98 µm and center-to-center distances of 1.96 µm, and (I, R) line widths of 0.87 µm and center-to-center distances of 1.74 µm. Scale bar, 20 μm in Fig. S5, A and B, J and K. Scale bar, 10 μm in Fig. S5, C to E, and L to N. Scale bar, 3 μm in Fig. S5, F to I, and O to R.


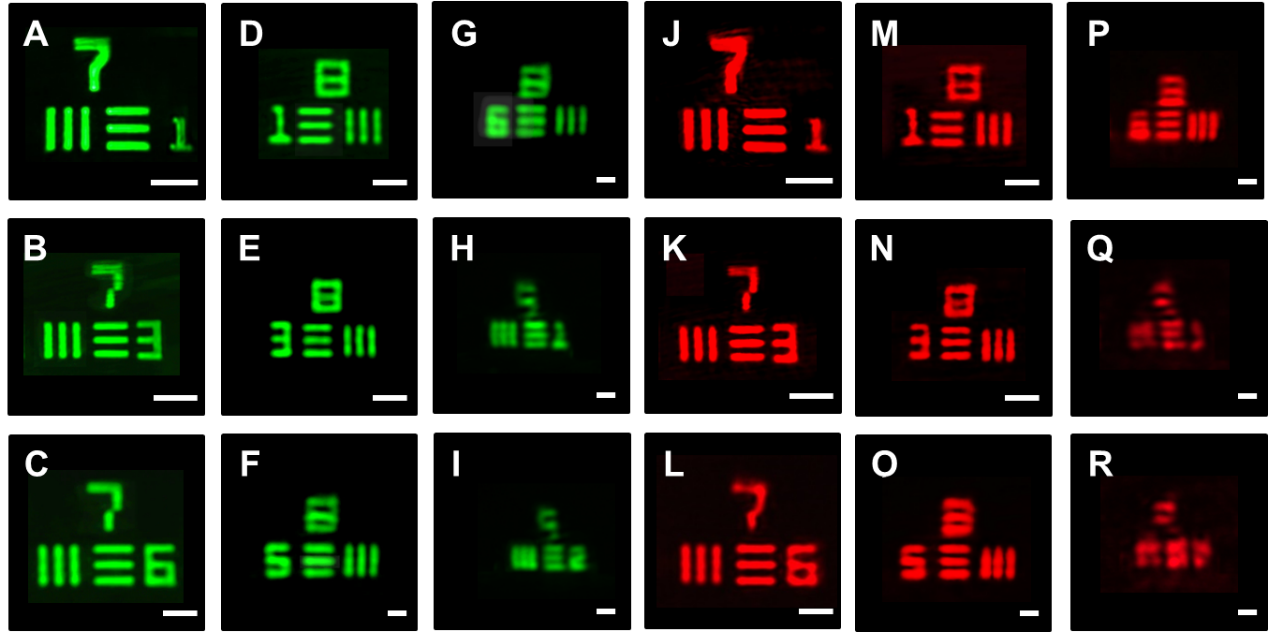


**Fig. S5: Images of the 1951 USAF resolution test chart formed by the metalenses at the laser wavelengths of 532 nm and 633nm.**
